# Barriers to Utilizing Breast Cancer Screening Methods Among Adult Females in Taif, Saudi Arabia: A Cross-Sectional Study

**DOI:** 10.7759/cureus.98966

**Published:** 2025-12-11

**Authors:** Sahar Alnefaie, Suhaiyh S Alotibi, Manar D Almutairi, Najla M Alharthi, Fahad S Altowairqi, Naif F Alahmari

**Affiliations:** 1 Department of Surgery, College of Medicine, Taif University, Taif, SAU; 2 Department of Emergency Medicine, Ministry of National Guard-Health Affairs, Jeddah, SAU; 3 Department of Surgery, King Abdullah Medical Complex, Jeddah, SAU

**Keywords:** breast cancer, breast cancer screening, clinical breast examination, screening mammogram, self-breast examination

## Abstract

Introduction

Breast cancer (BC) is one of the leading causes of cancer-related deaths worldwide. Screening methods for early detection have significantly reduced mortality rates and improved survival rates associated with this disease. The study presented in this paper aimed to identify the barriers to regular breast cancer screening methods for early detection among adult females in Taif City.

Objectives

This analytical cross-sectional study sought to identify the most prevalent barriers to the use of various breast cancer screening methods.

Methods

A cross-sectional study was conducted between January 2024 and November 2024. A validated and pretested questionnaire was utilized for data collection after identifying the participants, who were women aged 18 years and older, residing in Taif City. An online questionnaire was sent out to these 405 females. The questionnaire comprised questions on the participants’ socio-demographic details, breast cancer-related background, and screening-related aspects that covered personal experiences, including those related to routine clinical breast examinations (CBEs), breast self-examinations (BSEs), and mammography. Pertinent questions-drawing on the modified champion health beliefs model scale (CHBMS)-were also included.

Results

Of the 405 women respondents from Taif, 4.7% reported undergoing mammography regularly, 7.9% indicated they undergo CBE regularly, and 25.7% stated they perform BSE regularly. The most commonly reported warning sign of breast cancer among participants was pain in one breast or axilla, noted by 73.3%. The most frequently identified risk factors were a previous history of breast cancer (67.7%) and having a close relative who was diagnosed with breast cancer (63.3%). The primary barrier to BSE was difficulty remembering to perform the examination, reported by 42.2% of participants. Conversely, the main barrier to CBE was embarrassment, cited by 37.3% of participants. The primary barriers preventing women from undergoing mammography were embarrassment (30.4%) and pain (17.8%).

Conclusion

Participants demonstrated a high level of awareness regarding breast cancer symptoms and risk factors; however, overall screening rates remain low. The study revealed several barriers preventing women from undergoing screening, including anxiety about BSE and some feeling embarrassed as regards CBE. There is, thus, an urgent need for a comprehensive approach to educate women about the importance of breast cancer screening. Educational programs to enhance awareness and encourage early detection are the need of the hour.

## Introduction

Breast cancer is a leading cause of death among women, and early detection has been linked to decreased morbidity and mortality associated with this disease [1]. In 2020, breast cancer affected 2.3 million women and resulted in 685,000 fatalities worldwide, with 2,484 cases reported among adults in Saudi Arabia. By the end of that year, there were 7.8 million women who had survived breast cancer within the previous five years, making it the most prevalent form of cancer globally [2].

A recent study indicated that the increased incidence of breast cancer in countries with a higher Human Development Index (HDI) is attributed to long-standing reproductive and hormonal risk factors (early onset of menstruation, delayed menopause, older age at first childbirth, fewer pregnancies, lack of breastfeeding, menopausal hormone therapy, and use of oral contraceptives) as well as lifestyle risk factors (alcohol consumption, obesity, and lack of physical activity) [3,4].

Early detection of breast cancer and timely treatment are crucial methods to prevent fatalities caused by this disease. The most reliable approach to identifying breast cancer at an early stage is through regular screening tests [5]. The primary diagnostic methods recommended by the World Health Organization are CBE and mammography. Where CBE and mammography are difficult or impossible to avail as options, BSE is suggested as a secondary tool. It is essential for women to regularly undergo mammography and physical breast exams whether done by healthcare professionals or themselves. Women should be familiar with the normal appearance and texture of their breasts to identify any changes [5,6].

There is a paucity of research in Saudi Arabia regarding knowledge, attitudes, and behaviors related to breast cancer. One study, however, observed a higher level of awareness about breast cancer among older women residing in Riyadh. Out of 864 women aged between 20 and 50 years who were involved in this study, 82% were aware of BSE, and 61% had knowledge about mammography. Surprisingly, however, only 41.2% reported having done BSE(s), while only 18.2% had ever undergone a mammography [7].

A study conducted in Al Hassa governorate found that none of the participants reported having mammography as part of their age-specific screening. The majority had decided to undergo a procedure based on what their healthcare provider advised. Mammography was performed for diagnostic purposes in 50.5% of cases, and 32.1% of the 50 years or older participants underwent it as a follow-up [8]. A different study of teachers in their thirties indicated a limited understanding of breast cancer, with only 32.4% knowing about BSE [9].

A study conducted in Jeddah involving 328 participants revealed that the primary obstacle for BSEs was the fear of discovering that something was wrong with their breasts (47%). Similarly, embarrassment was the main hindrance to CBE (45.9%). Conversely, women cited embarrassment (36%) and pain (32.6%) as the main barriers preventing them from undergoing mammography [10].

The present study aimed to evaluate the obstacles that prevent adult females in Taif City, Saudi Arabia, from utilizing breast cancer screening methods.

## Materials and methods

Study design and settings 

This analytical cross-sectional study was conducted from January 2024 to November 2024 using a pre-tested and validated questionnaire, obtained from a previous research project. It was used without modification and translated into Arabic [11].

Study population 

The study included all women aged 18 years and older, residing in Taif City, according to the General Authority for Statistics [12].

Ethical considerations 

The study received ethics approval from the Research Ethics Committee at Taif University, Kingdom of Saudi Arabia, IRB approval number (45-345). Adult females in Taif City received the survey questionnaire via social media platforms such as WhatsApp and Snapchat. The participants were informed about the purpose of the study, and the data of only those who provided consent for their data to be used for the study’s purposes were included in the analysis.

Questionnaire structure and content

The questionnaire was divided into three parts (Appendix A, B, C). Part A had questions about the socio-demographic details of the participants, such as age, educational level, and marital status. Part B focused on knowing about the participants’ breast cancer background and screening, including personal experiences and whether a family member or friend was diagnosed with BC. It also assessed participants’ knowledge about warning signs, risk factors, and screening practices such as CBE, BSE, and mammography. Part C contained 22 multiple-choice questions (agree, neutral, or disagree), which recorded participants’ responses according to the modified Champion Health Belief Model Scale (CHBMS) questionnaire, the 22-item Modified Champion Health Belief Model Scale (CHBMS-BC-M) for assessing beliefs toward breast cancer screening. Before data collection, a pilot study involving 20 participants was conducted to refine survey questions and ensure the questionnaire's clarity, consistency, and validity.

Sample size calculation 

A sample size of 405 was calculated using the Epi Info application [13].

Statistical analysis 

The statistical analysis of data was performed using SPSS (IBM Corp., 2019, IBM SPSS Statistics for Windows, Version 26.0). The Chi-squared test (χ²) was applied to the qualitative data expressed in numbers and percentages to determine the association between the variables. Multivariable logistic regression analysis was conducted to assess the risk factors (independent predictors) vis-à-vis any barriers to regular BSE, CBE, or mammography. The odds ratio (OR) was calculated at a 95% confidence interval (CI) to evaluate these risk factors. A p-value of less than 0.05 was considered statistically significant.

## Results

Of the 405 participants, 298 (73.9%) were aged 20-29 years, 286 (70.6%) were single, and 260 (64.2%) had a college or university education. Further, 97 (24.0%) had a family member or friend who was diagnosed with breast cancer, 11 (2.7%) had been diagnosed with breast cancer, and 292 (72.1%) and 229 (56.5%) reported knowledge about breast cancer’s warning signs and risk factors, respectively. Only 19 (4.7%), 32 (7.9%), and 104 (25.7%) had undergone regular mammography, CBEs, and BSEs, respectively (Table 1).

**Table 1:** Distribution of study participants based on their demographic characteristics, BC history, BC knowledge, and whether they were regularly tested using any of the screening tests (N=405). BC: breast cancer.

Variable	N (%)
Age	
20-29	298 (73.9)
30-39	40 (9.9)
40-44	29 (7.2)
≥ 45	38 (9.4)
Marital status	
Widower	2 (0.5)
Single	286 (70.6)
Married	105 (25)
Divorced	12 (3)
Educational level	
Illiterate	3 (0.7)
Primary	10 (2.5)
Intermediate	9 (2.2)
Secondary	123 (30.4)
University or college and above	260 (64.2)
Do you have a family member or friend experienced breast cancer?	
No	208 (76)
Yes	97 (24)
Have you ever been diagnosed with breast cancer?	
No	394 (97.3)
Yes	11 (2.7)
Do you know any of the warning signs of breast cancer?	
No	113 (27.9)
Yes	292 (72.1)
Do you know any of the risk factors of breast cancer?	
No	176 (43.5)
Yes	229 (56.5)
Do you undergo mammography on a regular basis (if you are over 40?)	
I am less than 40 years old	294 (72.6)
No	92 (22.7)
Yes	19 (4.7)
Do you have CBE on a regular basis?	
No	373 (92.1)
Yes	32 (7.9)
Do you have breast self-examinations on a regular basis?	
No	301 (74.3)
Yes	104 (25.7)

**Figure 1:**
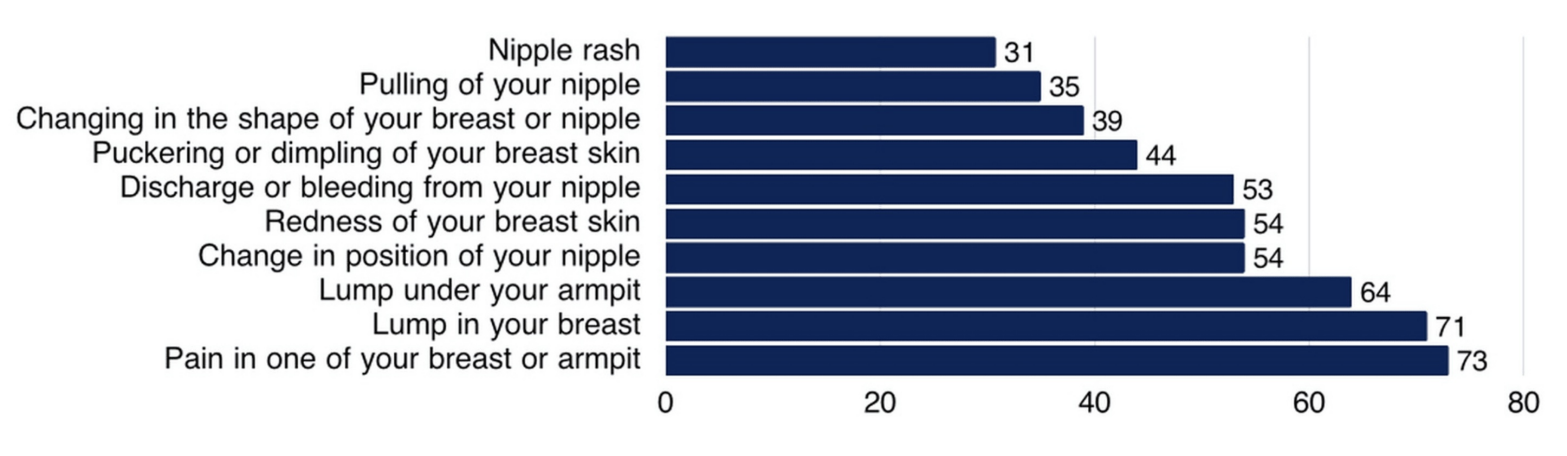
Percentage distribution of participants’ knowledge about BC warning signs (N=405). BC: breast cancer.

The most commonly recognized BC warning signs included pain in one of the breasts or armpits (297, 73.3%), a lump in the breast (287, 70.9%), and a lump under the armpit (261, 64.4%), as illustrated in Figure 1.

**Figure 2:**
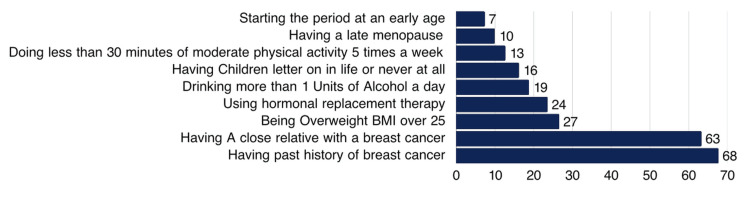
Percentage distribution of participants’ knowledge about BC risk factors (N=405). BC: breast cancer.

Having a history of breast cancer (274, 67.7%) and a close relative who was diagnosed with breast cancer (256, 63.3%) were considered the most significant risk factors (Figure 2).

Of the 405 participants, 171 (42.2%) reported that it is hard to remember to perform BSE, 24 (5.9%) believed they were likely to get breast cancer, and 28 (6.9%) perceived a high likelihood of developing BC within the next few years. Additionally, 192 (47.4%) expressed that the thought of breast cancer scares them, and 158 (39%) reported that having breast cancer would change one’s whole life.

Regarding barriers to BSE, 163 (40.2%) felt that doing BSE would make them worry about what is wrong with their breast, and 61 (15.1%) thought that BSE takes too much time. Concerning CBE barriers, 151 (37.3%) found it embarrassing to have a breast exam performed by a physician, 82 (20.2%) believed that breast exams are painful, 75 (18.5%) were afraid they would not be able to go to a physician for a breast exam, and 87 (21.5%) considered it time-consuming. Furthermore, 70 (17.3%) reported having other problems more important than getting a mammogram done, 72 (17.8%) said that mammography is too painful, 123 (30.4%) found it too embarrassing, 63 (15.6%) were unable to turn up for their mammography appointment, and 69 (17%) believed that getting a mammogram done takes too much time.

Regarding participants’ confidence in BSE’s efficacy, 143 (35.2%) reported that they could find a breast lump by performing BSE, 165 (40.7%) stated they could tell if something was wrong with their breasts when looking at them in the mirror, and 130 (32.2%) felt they could perform BSE correctly.

Health motivations included exercising at least three times per week (124, 30.6%), eating well-balanced meals (159, 39.3%), and maintaining good health, being extremely important to them (291, 71.9%) (Table 2).

**Table 2:** Responses from participants to the modified CHBMS questionnaire. CHBMS: champion health beliefs model scale.

Concept	Disagree	Neutral	Agree
	N (%)	N (%)	N (%)
Susceptibility			
It is likely that I will get breast cancer	232 (57.3)	149 (36.8)	24 (5.9)
My chances of getting breast cancer in the next few years are great	254 (62.7)	123 (30.4)	28 (6.9)
Seriousness			
The thought of breast cancer scares me	97 (24)	116 (28.6)	192 (47.4)
If someone had breast cancer, her whole life would change	102 (25.2)	145 (35.8)	158 (39)
Barriers			
BSE barriers: doing breast examination will make me worry about what is wrong with my breast	117 (28.9)	125 (30.9)	163 (40.2)
BSE takes too much time	188 (46.4)	156 (38.5)	61 (15.1)
It is hard to remember to do a breast examination	102 (25.2)	132 (32.6)	171 (42.2)
CBE barriers: It is embarrassing for me to have a breast exam performed by a physician	126 (31.1)	128 (31.6)	151 (37.3)
Breast exams performed by a physician can be painful	185 (45.7)	138 (34.1)	82 (20.2)
I am afraid I would not be able to go for a breast exam performed by a physician.	209 (51.6)	121 (29.9)	75 (18.5)
Breast exam performed by a physician is time-consuming	160 (39.5)	158 (39)	87 (21.5)
Mammography barriers: I have other problems more important than getting a mammogram	171 (42.2)	164 (40.5)	70 (17.3)
Having a mammogram is too painful	188 (46.4)	145 (35.8)	72 (17.8)
I am afraid I would not be able to go to the mammogram appointment	200 (49.4)	142 (35.1)	63 (15.6)
Having a mammogram is too embarrassing	151 (37.3)	131 (32.3)	123 (30.4)
Having a mammogram takes too much time	156 (38.5)	180 (44.4)	69 (17)
Confidence in BSE efficacy			
I could find a breast lump by performing BSE	97 (24)	166 (41)	142 (35.2)
I can tell that something is wrong with my breast when I look in the mirror	93 (23)	147 (36.3)	165 (40.7)
I can perform BSE correctly	120 (29.6)	154 (38)	131 (32.3)
Health motivation			
I exercise at least three times/week	166 (41)	115 (28.4)	124 (30.6)
I eat well-balanced meals	89 (22)	157 (38.8)	159 (39.3)
Maintaining good health is extremely important to me	32 (7.9)	82 (20.2)	291 (71.9)

**Figure 3:**
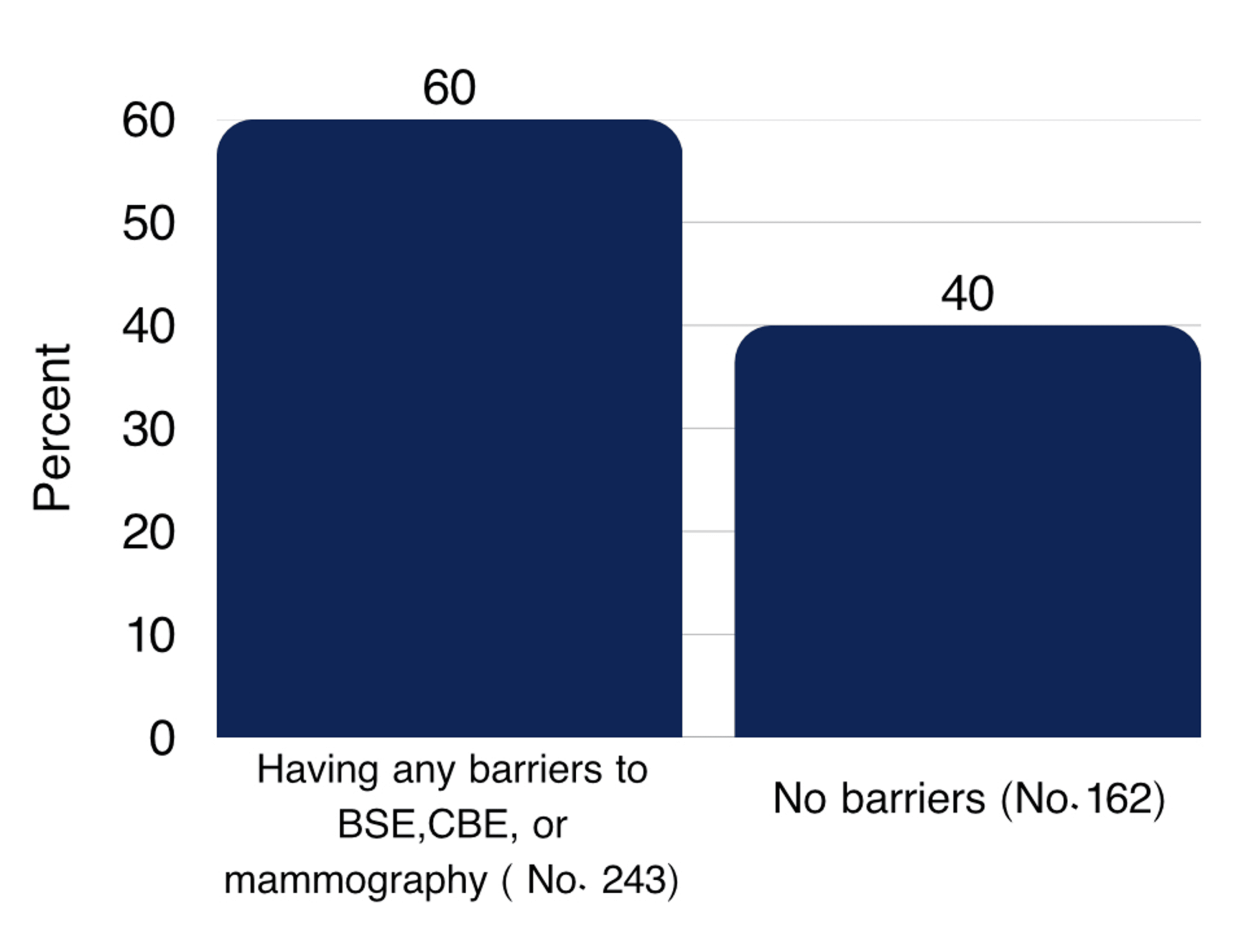
Prevalence of barriers to BSE, CBE, or mammography. BSE: breast self examination, CBE: clinical breast examination.

243 (60%) of the participants faced barriers to getting BSE, CBE, or mammography done (Figure 3).

Although the prevalence of barriers to BSE, CBE, or mammography was higher among those aged 20-29 years, being single, having had higher education, not having a family member or friend who was diagnosed with breast cancer, and never being diagnosed with breast cancer were not significantly related to the prevalence of barriers to BSE, CBE, and mammography (p< 0.05). Similarly, no significant relationship was found between the prevalence of barriers to BSE, CBE, or mammography and participants’ knowledge of BC warning signs or risk factors or their regular participation in BC screening tests (p< 0.05) (Table 3).

**Table 3:** Relationship between the prevalence of barriers to BSE, CBE, or mammography and participants’ demographic characteristics, BC history, BC knowledge, and their regular BC screening tests (N=405). BSE: breast self examination, CBE: clinical breast examination, BC: breast cancer.

Variable	Having any barrier to BSE, CBE, or mammography		χ2	p-value
	Yes, N (%)	No, N(%)		
Age				
20-29	173 (71.2)	125 (77.2)	2.06	0.56
30-39	26 (10.7)	14 (8.6)		
40-44	18 (7.4)	11 (6.8)		
≥ 45	26 (10.7)	12 (7.4)		
Marital status				
Widower	2 (0.8)	0 (0.0)	4.13	0.247
Single	164 (67.5)	122 (75.3)		
Married	70 (28.8)	35 (21.6)		
Divorced	7 (2.9)	5 (3.1)		
Educational level				
Illiterate	0 (0.0)	3 (1.9)	7.33	0.119
Primary	8 (3.3)	2 (1.2)		
Intermediate	6 (2.5)	3 (1.9)		
Secondary	78 (32.1)	45 (27.8)		
University or college and above	151 (62.1)	109 (67.3)		
Do you have a family member or friend experienced breast cancer?				
No	180 (74.1)	128 (79)	1.3	0.254
Yes	63 (25.9)	34 (21)		
Have you ever been diagnosed with breast cancer?				
No	235 (96.7)	159 (98.1)	0.76	0.382
Yes	8 (3.3)	3 (1.9)		
Do you know any of the warning signs of breast cancer?				
No	71 (29.2)	42 (25.9)	0.52	0.469
Yes	172 (70.8)	120 (74.1)		
Do you know any of the risk factors of breast cancer?				
No	98 (40.3)	78 (48.1)	2.41	0.12
Yes	145 (59.7)	84 (51.9)		
Do you undergo mammography on a regular basis (if you are over 40)?				
I am less than 40 years old	175 (72)	119 (73.5)	0.13	0.934
No	56 (23)	36 (22.2)		
Yes	12 (4.9)	7 (4.3)		
Do you have CBE on a regular basis?				
No	225 (92.6)	148 (91.4)	0.2	0.652
Yes	18 (7.4)	14 (8.6)		
Do you have breast self-examinations on a regular basis?				
No	180 (74.1)	121 (74.7)	0.01	0.889
Yes	63 (25.9)	41 (25.3)		

Among the participants over 40 years, those aged 45 years or more, those married, those not diagnosed with BC, and those who had CBE or BSE regularly (p< 0.05) were quite regular in undergoing mammography (Table 4).

**Table 4:** Relationship between undergoing mammography regularly and participants’ demographic characteristics, BC history, BC knowledge, and their regular BC screening tests (N=405). BC: breast cancer.

Variable	Do you undergo mammography on a regular basis (if you are over 40?)		χ2	p-value
	No, N (%)	Yes, N (%)		
Age				
20-29	293 (75.9)	5 (26.3)	39.44	<0.001
30-39	39 (10.1)	1 (5.3)		
40-44	24 (6.2)	5 (26.3)		
≥ 45	30 (7.8)	8 (42.1)		
Marital status				
Widower	1 (0.3)	1 (5.3)	35.4	<0.001
Single	283 (73.3)	3 (15.8)		
Married	92 (23.8)	13 (68.4)		
Divorced	10 (2.6)	2 (10.5)		
Educational level				
Illiterate	2 (0.5)	1 (5.3)	7.38	0.117
Primary	9 (2.3)	1 (5.3)		
Intermediate	8 (2.1)	1 (5.3)		
Secondary	117 (30.3)	6 (31.6)		
University or college and above	250 (64.8)	10 (52.6)		
Do you have a family member or friend experienced breast cancer?				
No	297 (76.9)	11 (57.9)	3.6	0.058
Yes	89 (23.1)	8 (42.1)		
Have you ever been diagnosed with breast cancer?				
No	382 (99)	12 (63.2)	17.86	<0.001
Yes	4 (1)	7 (36.8)		
Do you know any of the warning signs of breast cancer?				
No	110 (28.5)	3 (15.8)	1.45	0.228
Yes	276 (71.5)	16 (84.2)		
Do you know any of the risk factors of breast cancer?				
No	168 (43.5)	8 (42.1)	0.01	0.903
Yes	218 (56.5)	11 (57.9)		
Do you have CBE on a regular basis?				
No	365 (94.6)	8 (42.1)	18.47	<0.001
Yes	21 (5.4)	11 (57.9)		
Do you have breast self-examinations on a regular basis?				
No	296 (76.7)	5 (26.3)	24.06	<0.001
Yes	90 (23.3)	14 (73.7)		

The incidence of regular CBE was significantly higher among the participants aged 20-29 years, those who were single, those diagnosed with breast cancer, or those who performed BSEs regularly (p< 0.05) (Table 5).

**Table 5:** Relationship between undergoing CBEs regularly and participants’ demographic characteristics, BC history, BC knowledge, and their regular BC screening tests (N=405). CBE: clinical breast examination, BC: breast cancer.

Variable	Do you have CBE on a regular basis?		χ2	p-value
	No, N (%)	Yes, N (%)		
Age				
20-29	282 (75.6)	16 (50)	10.51	0.015
30-39	35 (9.4)	5 (15.6)		
40-44	24 (6.4)	5 (15.6)		
≥ 45	32 (8.6)	6 (18.8)		
Marital status				
Widower	1 (0.3)	1 (3.1)	13.52	0.004
Single	271 (72.7)	15 (46.9)		
Married	90 (24.1)	15 (46.9)		
Divorced	11 (2.9)	1 (3.1)		
Educational level				
Illiterate	2 (0.5)	1 (3.1)	5.65	0.227
Primary	8 (2.1)	2 (6.3)		
Intermediate	8 (2.1)	1 (3.1)		
Secondary	112 (30)	11 (34.4)		
University or college and above	243 (65.1)	17 (53.1)		
Do you have a family member or friend experienced breast cancer?				
No	283 (75.9)	25 (78.1)	0.08	0.774
Yes	90 (24.1)	7 (21.9)		
Have you ever been diagnosed with breast cancer?				
No	366 (98.1)	28 (87.5)	12.58	<0.001
Yes	7 (1.9)	4 (12.5)		
Do you know any of the warning signs of breast cancer?				
No	104 (27.9)	9 (28.1)	0.001	0.977
Yes	269 (72.1)	23 (71.9)		
Do you know any of the risk factors of breast cancer?				
No	160 (42.9)	16 (50)	0.6	0.437
Yes	213 (57.1)	16 (50)		
Do you have breast self-examinations on a regular basis?				
No	295 (79.1)	6 (18.8)	16.22	<0.001
Yes	78 (20.9)	26 (81.3)		

The incidence of regular BSE was significantly higher among the participants aged 20-29 years, those who were single, those with a family member or friend who was diagnosed with breast cancer, or those diagnosed with BC (p< 0.05) (Table 6).

**Table 6:** Relationship between doing BSEs regularly and participants’ demographic characteristics, BC history, BC knowledge, and their regular BC screening tests (N=405). BSE: breast self examination, CBE: clinical breast examination, BC: breast cancer.

Variable	Do you have breast self-examinations on a regular basis?		χ2	p-value
	No, N (%)	Yes, N (%)		
Age				
20-29	232 (77.1)	66 (63.5)	11.31	0.01
30-39	30 (10)	10 (9.6)		
40-44	16 (5.3)	13 (12.5)		
≥ 45	23 (7.6)	15 (14.4)		
Marital status				
Widower	1 (0.3)	1 (1)	14.71	0.002
Single	226 (75.1)	60 (57.7)		
Married	69 (22.9)	36 (34.6)		
Divorced	5 (1.7)	7 (6.7)		
Educational level				
Illiterate	2 (0.7)	1 (1)	2.41	0.659
Primary	8 (2.7)	2 (1.9)		
Intermediate	6 (2)	3 (2.9)		
Secondary	97 (32.2)	26 (25)		
University or college and above	188 (62.5)	72 (69.2)		
Do you have a family member or friend experienced breast cancer?				
No	239 (79.4)	69 (66.3)	7.23	0.007
Yes	62 (20.6)	35 (33.7)		
Have you ever been diagnosed with breast cancer?				
No	297 (98.7)	97 (93.3)	8.53	0.003
Yes	4 (1.3)	7 (6.7)		
Do you know any of the warning signs of breast cancer?				
No	87 (28.9)	26 (25)	0.58	0.444
Yes	214 (71.1)	78 (75)		
Do you know any of the risk factors of breast cancer?				
No	128 (42.5)	48 (46.2)	0.41	0.52
Yes	173 (57.5)	56 (53.8)		

Multivariable logistic regression analysis was performed to identify risk factors associated with barriers to regular BSE, CBE, or mammography among the study participants. None of the studied variables were found to be independent predictors of the barriers to regular BSE, CBE, or mammography (p< 0.05). However, for undergoing mammography regularly, older age (≥ 45 years), previous diagnosis of breast cancer, or undergoing CBEs regularly were identified as independent predictors (p< 0.05). An independent predictor for undergoing CBEs regularly was also doing BSEs regularly (p< 0.05). Further, the independent predictors for doing BSEs regularly were having a higher level of education (university or college and above), having a family member or friend who was diagnosed with breast cancer, or undergoing CBEs regularly (p< 0.05) (Tables 7, 8, 9, 10).

**Table 7:** Multivariable logistic regression analysis of risk factors concerning barriers to regular BSE, CBE, or mammography and undergoing mammography on a regular basis (N=405). For barriers to regular BSE, CBE, or mammography Model fit indices:* Nagelkerke R²=0.24; Hosmer–Lemeshow χ²=7.12, p=0.48; Predictive Accuracy Rate (PAR)=77.8%. For undergoing mammography on a regular basis, Model fit indices:* Nagelkerke R²=0.31; Hosmer–Lemeshow χ²=6.94, p=0.45; Predictive Accuracy Rate (PAR)=83.6%.

Variable	Category for OR calculation	p-value*	Odds ratio	Lower-upper (CI:95%)
Age	Continuous variable- odds decrease with age	0.44	0.88	(0.65-1.2)
Marital status	Married vs. single	0.906	0.96	(0.57-1.63)
Educational level	University vs. below	0.633	1.07	(0.79-1.47)
Do you have a family member or friend experienced breast cancer?	Yes vs. no	0.294	0.76	(0.46-1.26)
Have you ever been diagnosed with breast cancer?	Yes vs. no	0.606	0.66	(0.14-3.13)
Do you know any of the warning signs of breast cancer?	Yes vs. no	0.301	1.27	(0.8-2.01)
Do you know any of the risk factors of breast cancer?	Yes vs. no	0.077	0.68	(0.45-1.04)
Do you undergo mammography on a regular basis ?	Yes vs. no	0.863	1.11	(0.33-3.74)
Do you have CBE on a regular basis?	Yes vs. no	0.558	1.29	(0.54-3.05)
Do you have breast self-examinations on a regular basis?	Yes vs. no	0.915	0.97	(0.58-1.62)

**Table 8:** Multivariable logistic regression, undergoing mammography on a regular basis (N=405). For barriers to regular BSE, CBE, or mammography Model fit indices:* Nagelkerke R²=0.24; Hosmer–Lemeshow χ²=7.12, p=0.48; Predictive Accuracy Rate (PAR)=77.8%. For undergoing mammography on a regular basis, Model fit indices:* Nagelkerke R²=0.31; Hosmer–Lemeshow χ²=6.94, p=0.45; Predictive Accuracy Rate (PAR)=83.6%.

Variable	Category for OR calculation	p-value*	Odds ratio	Lower-upper (CI:95%)
Age	Continuous variable-odds increase with age	0.002	2.84	(1.45-5.56)
Marital status	Married vs. single	0.72	1.25	(0.36-4.22)
Educational level	University vs. below	0.705	1.16	(0.53-2.5)
Do you have a family member or friend experienced breast cancer?	Yes vs. no	0.833	0.85	(0.18-3.84)
Have you ever been diagnosed with breast cancer?	Yes vs. no	< 0.001	9.45	(2.59-5.67)
Do you know any of the warning signs of breast cancer?	Yes vs. no	0.279	2.47	(0.47-12.77)
Do you know any of the risk factors of breast cancer?	Yes vs. no	0.791	1.22	(0.26-5.65)
Do you have CBE on a regular basis?	Yes vs. no	< 0.001	9.5	(4.1-8.75)
Do you have breast self-examinations on a regular basis?	Yes vs. no	0.197	2.64	(0.6-2.54)

**Table 9:** Multivariable logistic regression analysis of factors associated with undergoing clinical breast examination on a regular basis (N=405). Model fit indices:* Nagelkerke R²=0.29; Hosmer–Lemeshow χ²=6.85, p=0.42; Predictive Accuracy Rate (PAR)=81.3%.

Variable	Category for OR calculation	p-value	Odds ratio	95% CI
Age	Continuous variable-odds increase with age	0.299	1.29	0.79-2.12
Marital status	Married vs. single	0.77	0.87	0.35-2.17
Educational level	University vs. below	0.154	0.67	0.38-1.16
Family member/friend experienced breast cancer	Yes vs. no	0.114	0.41	0.14-1.23
Previous diagnosis of breast cancer	Yes vs. no	0.06	0.32	0.93-3.4
Knowledge of warning signs	Yes vs. no	0.718	0.34	0.33-2.11
Knowledge of risk factors	Yes vs. no	0.689	0.83	0.34-2
Undergo mammography regularly	Yes vs. no	<0.001	9.5	4.1-8.75
Breast self-examinations on a regular basis	Yes vs. no	<0.001	2.57	2.68-4.68

**Table 10:** Multivariable logistic regression analysis of factors associated with having breast self-examination on a regular basis (N=405). Model fit indices:* Nagelkerke R²=0.29; Hosmer–Lemeshow χ²=6.85, p=0.42; Predictive Accuracy Rate (PAR)=81.3%.

Variable	Category for OR calculation	p-value	Odds ratio (OR)	95% Confidence interval (CI)
Age	Continuous variable -odds increase with age	0.43	1.14	0.81-1.62
Marital status	Married vs. single	0.17	1.50	0.83-2.70
Educational level	University vs. below	0.019	1.62	1.08-2.42
Family member or friend with breast cancer	Yes vs. no	0.018	1.95	1.11-3.41
Ever diagnosed with breast cancer	Yes vs. no	0.566	1.62	0.31-1.51
Knowledge of the warning signs of breast cancer	Yes vs. no	0.462	1.24	0.69-2.21
Knowledge of risk factors of breast cancer	Yes vs. no	0.477	0.82	0.49-1.38
Regular mammography	Yes vs. no	0.197	2.64	0.60-2.54
Regular clinical breast examination (CBE)	Yes vs. no	<0.001	2.57	2.68-4.68

## Discussion

The present study aimed to identify the barriers that prevent adult women in Taif from undergoing regular breast cancer screenings. It focused on the most common obstacles that hinder the use of various screening methods. The study also examined the social and demographic factors influencing women’s decisions regarding screening. The results indicated that participants had a good level of awareness about BC, including its symptoms and risk factors. A study conducted in Jeddah, which has been referred to earlier, also found that the women participants had a strong understanding of BC [10]. In contrast, a study conducted on female patients at primary healthcare centres in Najran revealed that more than half of the participants had a low level of knowledge about BC and screening methods [14].

Despite the noticeable increase in knowledge about breast cancer and various screening methods, such awareness remains insufficient to facilitate early detection and treatment.

The present study highlights significant awareness among participants regarding the barriers to BC. The results of the present study showed that several barriers affected the ability of women in Taif City to undergo screening; half of the participants expressed anxiety about performing BSE(s), while the majority felt embarrassed about undergoing CBE(s) conducted by a physician. Regarding screening for a mammogram, most participants did not report any specific barriers that prevented them from undergoing mammography.

Previous studies have shown regional differences in awareness. A study conducted in Oman found that the most common barriers related to BCs were personal, including embarrassment about breast exams, fear of a cancer diagnosis, and concerns about side effects from treatments like mastectomy, chemotherapy, and radiation [15]. Similarly, a study of Palestinian women in the West Bank identified a mix of cultural and personal barriers to screening, such as limited medical facilities, cost and accessibility issues, inaccurate knowledge, and fear of diagnosis or treatment side effects [16]. Additionally, a recent study in Saudi Arabia highlighted the fear of diagnosis, embarrassment, and lack of awareness as major barriers to BCs [17]. These findings emphasize the impact of demographic and professional factors on awareness, suggesting that the recent increase in local percentages indicates a gradual and promising rise in public awareness.

The present study found that undergoing mammography was higher among women aged 45 and older, married women, and those with higher educational levels. Similar findings in other studies reported an association between sociodemographic factors and the likelihood of undergoing mammography [18]. However, the present study’s results indicate limited recourse to mammography in the Taif region despite the availability of free health services. According to a study of Saudi women aged 50 years or older, the low rate of opting for mammography is not unique to the Taif region [19]. Our research shows that having a family member who was diagnosed with BC is a factor that encourages women to undergo mammography regularly. Some other studies concerning women in Saudi Arabia reported contrary results [20]. Hence, we must enhance awareness and knowledge about BC risk factors in our community. More than half of the participants who undergo mammography regularly also undergo CBE and do BSE, indicating their concern about BC and attention to personal health.

Regarding CBE, almost half of the women who undergo CBEs are between the ages of 20 and 29. This finding is similar to that of a study conducted in Yemen that reported limited practice of CBE among older women [21]. Further, more than half of the participants who undergo CBEs have higher educational qualifications. Thus, younger age, single marital status, and a higher level of education are strong factors associated with regular CBE. Moreover, women with a positive family history of BC reported lower rates of CBE compared to those without such a history, possibly due to a lack of knowledge about the importance of family history as a significant risk factor for BC. The aforementioned study addressing participants’ knowledge of BC risk factors in Oman also found that less than half of the participants believed they were at risk of BC if a relative was diagnosed with BC [15]. However, in general, most women who undergo CBEs and even those who do not have a good understanding of BC warning signs and risk factors.

Regular BSE was notably higher among younger, single women and those with a close personal experience of breast cancer, reflecting that the majority of respondents are aware of BSE [22]. The findings of the present study revealed that none of the studied variables were independently associated with barriers to doing BSEs and undergoing CBEs or mammography regularly. However, our results showed significant predictors for undergoing mammography, CBEs, and BSEs regularly, indicating that certain factors may play an influential role in motivating women toward regular breast cancer screening. Older age (≥ 45 years), previous breast cancer diagnosis, and regular CBE emerged as predictors for undergoing mammography regularly [10]. The present study also found that regular BSE was an independent predictor of regular CBE. Higher education, personal connections to breast cancer survivors, and regular CBE were all associated with doing BSEs regularly. Higher education often correlates with increased health awareness and access to health information, fostering proactive health behaviors. Additionally, knowing someone affected by breast cancer may raise awareness and perceived risk, promoting preventive actions.

Our study identified barriers that prevent adult women in Taif City from undergoing regular breast cancer screenings. Participants generally demonstrated a good awareness of symptoms and risk factors. However, the present study has some limitations that future research should address, such as the potential for bias due to its cross-sectional design, which precludes establishing causality. Additionally, some factors, such as the quality of services provided and the skills of healthcare providers, were not considered in the present study. Therefore, further research in this area should focus on examining these other important aspects to better understand them and address them as required.

## Conclusions

The present study showed that its female participants had a high level of awareness regarding breast cancer symptoms and risk factors, but this awareness did not translate into consistent screening behaviors. It also revealed several barriers affecting the ability to undergo screening: half of the participants felt anxious about performing BSE, while the majority felt embarrassed about undergoing CBE. While some women reported embarrassment and pain as barriers to mammography, the majority did not report specific barriers. However, the overall screening rate in the region remains low despite the availability of health services. Additional efforts are needed to increase women’s awareness about the importance of breast cancer screening and encourage their participation in such screenings through targeted campaigns on social media, in shopping malls, and at universities. It is also essential to evaluate the quality of screening services to ensure they meet international standards, thereby supporting effective early detection and improving health outcomes.

## Appendices

Appendix A

Questionnaire

The survey used in this study was obtained from a previous research project without modifications [11].

Part A: socioeconomic details

**Table 11:** Socioeconomic details. The survey used in this study was obtained from a previous research project without modifications, permission was taken to use the questionnaire [11].

النص الأصلي )بالعربي)	Translation (English)
العمر: (20-29) (30-39) (40-44) (≥45)	Age (20-29); (30-39); (40-44); (≥45)
الحالة الاجتماعية: مطلقة (Divorced) متزوجة (Married) عزباء (Single) أرملة (Widower)	Marital status: Divorced; Married; Single; Widower
المستوى التعليمي: غير متعلمة (Illiterate) ابتدائي (Primary) متوسط (Intermediate) ثانوي (Secondary) جامعة / كلية او دراسات عليا (University or college & above)	Educational level: Illiterate; Primary; Intermediat; Secondary; University or college and above

Appendix B

Questionnaire

Part B: knowing about the participants’ breast cancer background and screening, including personal experiences and whether a family member or friend was diagnosed with BC. It also assessed participants’ knowledge about warning signs, risk factors, and screening practices such as CBE, BSE, and mammography. 

**Table 12:** Knowing about the participants’ breast cancer background and screening, including personal experiences and whether a family member or friend was diagnosed with BC. It also assessed participants’ knowledge about warning signs, risk factors, and screening practices such as CBE, BSE, and mammography. The survey used in this study was obtained from a previous research project without modifications, permission was taken to use the questionnaire [11].

النص الأصلي )بالعربي)	Translation (English)
هل تعرض أحد أفراد عائلتك أو أحد صديقاتكللإصابة بسرطان الثدي؟ (نعم / لا)	Do you have a family member or friend who experienced breast cancer? (Yes/No)
هل لديك تاريخ مرضي سابق بالإصابة بسرطانالثدي؟ (نعم / لا)	Have you ever been diagnosed with breast cancer? (Yes/No)
هل تعرفين أي من علامات أو أعراض الإصابةبسرطان الثدي؟ (إذا كانت إجابتك بنعم، حددي أي من العلامات أوالأعراض التالية: تغير موضع الحلمة (نعم / لا) الحلمة الغارقة أو المقلوبة (نعم / لا) ألم في الإبط أو الثدي (نعم / لا) تغير في جلد الثدي (كالتنقير - مثل قشر البرتقالة) (نعم / لا) إفرازات من الحلمة (سائل شفاف أو دموي) (نعم / لا) وجود كتلة في الثدي (نعم / لا) ظهور طفح جلدي على الحلمة (نعم / لا) احمرار في جلد الثدي (نعم / لا) وجود كتلة في الإبط (نعم / لا) تغيرات في شكل الثدي أو الحلمة (نعم / لا	Do you know any of the warning signs of breast cancer? (If yes, please select the signs you know below) Change in position of your nipple (Yes/No); Pulling in of your nipple (Yes/No); Pain in one of your breasts or armpits (Yes/No); Puckering or dimpling of your breast skin (like orange peel) (Yes/No); Discharge or bleeding from your nipple (clear or bloody) (Yes/No); A lump in your breast (Yes/No); Nipple rash (Yes/No); Redness of your breast skin (Yes/No); A lump under your armpit (Yes/No); Changes in the shape of your breast or nipple (Yes/No).
هل تعرفين أي عوامل تزيد من نسبة الإصابة بسرطانالثدي؟ (إذا كانت إجابتك بنعم، حددي أي من العلامات أوالأعراض التالية تاريخ مرضي سابق بالإصابة بسرطان الثدي (نعم / لا) العلاج بالهرمونات البديلة (نعم / لا) تناول المشروبات التي تحتوي على الكحول (نعم / لا) الوزن الزائد (مقياس كتلة الجسم أعلى من 25) (نعم / لا) تاريخ عائلي بالإصابة بسرطان الثدي (نعم / لا) الإنجاب في سن متأخرة نسبياً / أو عدم الإنجاب (نعم / لا) الحيض في سن مبكرة نسبياً (نعم / لا) الوصول إلى سن اليأس في سن متأخرة نسبياً (نعم / لا) قلة النشاط البدني (أقل من 30 دقيقة من النشاط المعتدل 5 مرات في الأسبوع) (نعم / لا)	Do you know any of the risk factors of breast cancer? (If yes, please select the signs you know below.) Having a past history of breast cancer (Yes/No); Using HRT (Hormone Replacement Therapy) (Yes/No); Drinking more than 1 unit of alcohol a day (Yes/No); Being overweight (BMI over 25) (Yes/No); Having a close relative with breast cancer (Yes/No); Having children later in life or not at all (Yes/No); Starting your periods at an early age (Yes/No); Having a late menopause (Yes/No); Doing less than 30 mins of moderate physical activity 5 times a week (Yes/No);
هل تخضعين لتصوير الثدي بالأشعة السينية(الماموجرام) بشكل دوري (إذا كنتِ فوق عمر 40)؟(نعم / لا)	Do you undergo mammography on a regular basis (if you are over 40)? (Yes/No)
هل تخضعين لفحص وقائي للثدي من قبل مختصبشكل دوري؟ (نعم / لا)	Do you have CBE (clinical breast examination) on a regular basis? (Yes/No)
هل تقومين بإجراء فحص ذاتي للثدي بشكل دوري؟(نعم / لا)	Do you have breast self-examinations on a regular basis? (Yes/No)

Appendix C

Questionnaire

Part C: modified Champion's Health Belief Model Scale (CHBMS). The questionnaire consists of 22 items, and each item has four response options: Concept, Agree, Neutral, Disagree.

**Table 13:** Modified Champion's Health Belief Model Scale (CHBMS). The survey used in this study was obtained from a previous research project without modifications, permission taken to use the questionnaire [11].

النص الأصلي باللغة العربية	Translation
من المحتمل أن أصاب بسرطان الثدي. | أوافق | محايد | لا أوافق	It is likely that I will get breast cancer. | Agree | Neutral | Disagree
فرص إصابتي بسرطان الثدي في السنوات القليلة القادمة كبيرة. | أوافق | محايد | لا أوافق	My chances of getting breast cancer in the next few years are great. | Agree | Neutral | Disagree
فكرة الإصابة بسرطان الثدي تخيفني. | أوافق | محايد | لا أوافق	The thought of breast cancer scares me. | Agree | Neutral | Disagree
إذا أصيبت امرأة بسرطان الثدي، فإن حياتها كلها ستتغير. | أوافق | محايد | لا أوافق	If someone had breast cancer, her whole life would change. | Agree | Neutral | Disagree
إجراء فحص الثدي يجعلني أقلق بشأن ما قد يكون خطأ في ثديي. | أوافق | محايد | لا أوافق	Doing breast examination will make me worry about what is wrong with my breast. | Agree | Neutral | Disagree
فحص الثدي الذاتي يستغرق وقتًا طويلاً. | أوافق | محايد | لا أوافق	BSE takes too much time. | Agree | Neutral | Disagree
من الصعب أن أتذكر القيام بفحص الثدي. | أوافق | محايد | لا أوافق	It is hard to remember to do a breast examination. | Agree | Neutral | Disagree
أشعر بالحرج من إجراء فحص الثدي عند الطبيب. | أوافق | محايد | لا أوافق	It is embarrassing for me to have a breast exam performed by a physician. | Agree | Neutral | Disagree
فحص الثدي عند الطبيب قد يكون مؤلمًا. | أوافق | محايد | لا أوافق	Breast exams performed by a physician can be painful. | Agree | Neutral | Disagree
أخشى أنني قد لا أستطيع الذهاب لإجراء فحص الثدي عند الطبيب. | أوافق | محايد | لا أوافق	I am afraid I would not be able to go for a breast exam performed by a physician. | Agree | Neutral | Disagree
فحص الثدي عند الطبيب يستغرق وقتًا طويلاً. | أوافق | محايد | لا أوافق	Breast exam performed by a physician is time-consuming. | Agree | Neutral | Disagree
لدي مشاكل أخرى أكثر أهمية من إجراء تصوير الثدي (الماموجرام). | أوافق | محايد | لا أوافق	I have other problems more important than getting a mammogram. | Agree | Neutral | Disagree
إجراء تصوير الثدي (الماموجرام) مؤلم للغاية. | أوافق | محايد | لا أوافق	Having a mammogram is too painful. | Agree | Neutral | Disagree
أخشى أنني قد لا أستطيع الذهاب لموعد تصوير الثدي (الماموجرام). | أوافق | محايد | لا أوافق	I am afraid I would not be able to go to the mammogram appointment. | Agree | Neutral | Disagree
إجراء تصوير الثدي (الماموجرام) محرج للغاية. | أوافق | محايد | لا أوافق	Having a mammogram is too embarrassing. | Agree | Neutral | Disagree
إجراء تصوير الثدي (الماموجرام) يستغرق وقتًا طويلاً. | أوافق | محايد | لا أوافق	Having a mammogram takes too much time. | Agree | Neutral | Disagree
يمكنني اكتشاف وجود كتلة في الثدي عند القيام بفحص الثدي الذاتي. | أوافق | محايد | لا أوافق	I could find a breast lump by performing BSE. | Agree | Neutral | Disagree
أستطيع معرفة ما إذا كان هناك خطأ في ثديي عند النظر في المرآة. | أوافق | محايد | لا أوافق	I am able to tell something is wrong with my breast when I look in the mirror. | Agree | Neutral | Disagree
أستطيع القيام بفحص الثدي الذاتي بشكل صحيح. | أوافق | محايد | لا أوافق	I can perform BSE correctly. | Agree | Neutral | Disagree
أمارس الرياضة 3 مرات على الأقل في الأسبوع. | أوافق | محايد | لا أوافق	I exercise at least 3 times per week. | Agree | Neutral | Disagree
أتناول وجبات متوازنة. | أوافق | محايد | لا أوافق	I eat well-balanced meals. | Agree | Neutral | Disagree
الحفاظ على صحة جيدة أمر مهم للغاية بالنسبة لي. | أوافق | محايد | لا أوافق	Maintaining good health is extremely important to me. | Agree | Neutral | Disagree
